# Recent advances in the management of systemic lupus erythematosus

**DOI:** 10.12688/f1000research.13941.1

**Published:** 2018-06-29

**Authors:** Savino Sciascia, Massimo Radin, Dario Roccatello, Giovanni Sanna, Maria Laura Bertolaccini

**Affiliations:** 1 Center of Research of Immunopathology and Rare Diseases, Coordinating Center of Piemonte and Valle d’Aosta Network for Rare Diseases, Department of Clinical and Biological Sciences, and SCDU Nephrology and Dialysis, S. Giovanni Bosco Hospital, Turin, Italy; 2 Louise Coote Lupus Unit, Guy’s and St Thomas’ NHS Foundation Trust, London, UK; 3 Academic Department of Vascular Surgery, School of Cardiovascular Medicine & Sciences, King’s College London, London, UK

**Keywords:** biologics, B-cells, targeted therapy, clinical trials

## Abstract

Systemic lupus erythematosus (SLE) is a chronic autoimmune disease presenting highly heterogeneous clinical manifestations and multi-systemic involvement. Patients are susceptible to relapse­ and remission, thus making management challenging. Moreover, a considerable number of side effects may occur with conventional therapies; therefore, there is clearly a need for new therapeutic strategies. Since the pathogenesis of SLE is highly complex, it is far from being fully understood. However, greater understanding of the pathways and of the cellular and molecular mediators involved in SLE is being achieved. Emerging evidence has allowed the development of new biological therapeutic options targeting crucial molecular mediators involved in the pathogenesis of SLE. This literature review analyzes the availability of biological and target-directed treatments, phase II and III trials, and new therapies that are being developed for the treatment of SLE.

## Introduction

Systemic lupus erythematosus (SLE) is a chronic autoimmune disease characterized by relapses and flares with alternating periods of remission. The clinical manifestations are extremely heterogeneous with multi-systemic involvement, including symptoms such as fever and malaise, as well as dermatological, musculoskeletal, renal, respiratory, cardiovascular, hematological, and neurological manifestations ^1, 2^. Until recently, the treatment and management of SLE were based mainly on non-steroidal anti-inflammatory drugs, glucocorticoids, hydroxychloroquine, and immunosuppressive agents ^3^. Progress in the treatment of SLE has resulted in a significant improvement in prognosis. Nonetheless, SLE management is challenging because of the adverse effects of conventional therapies and the occurrence of refractory disease. Thus, the search for new therapeutic strategies is relentless. SLE may affect almost any organ during the disease course, and several pathogenic pathways drive SLE inflammation in affected tissues. Among other processes, the apoptotic process was thoroughly investigated; in particular, the crosslink among apoptotic debris-containing autoantigens, innate immunity activation, and the maintenance of inflammation has been further elucidated. Genes that breach immune tolerance and promote autoantibody production have also been investigated as part of the complex mosaic underlying SLE development, as they have been shown to influence innate immune signaling and type I interferon (IFN) production, which in turn can generate an influx of effector leukocytes, inflammatory mediators, and autoantibodies toward involved organs, such as the kidneys.

Besides, the investigation of monogenic forms of SLE over the years has triggered a better understanding of the SLE pathophysiological mechanisms. The findings that homozygous C1q deficiency and genetic mutations resulting in low levels of C2 and C4 significantly increase the risk of developing SLE are representative examples.

Given the broad heterogeneity of SLE with regard to genotype and clinical presentation, it is not surprising that there is no single drug that is able to improve all manifestations. A better understanding of SLE pathogenic mechanisms is well mirrored by some proposed synthetic drugs, such as tacrolimus, or biologics, including IFN-α inhibitors and other drugs capable of modulating the immune system.

**Figure 1.**
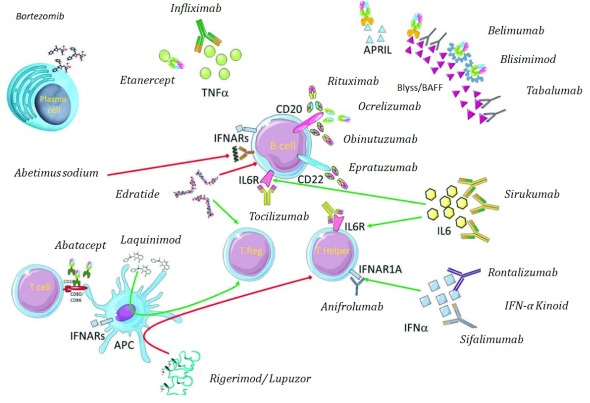
Targeted biological agents available and in ongoing phase II and III trials of systemic lupus erythematosus.

Attempts to reach a greater understanding of the underlying pathogenesis have resulted in the investigation of biological therapies that target crucial molecular mediators of SLE (as summarized in Figure 1). Biological therapy is emerging as an increasingly important treatment for autoimmune diseases, including SLE.

This literature review analyzes available data on biological and target-directed treatments, on phase II and III trials, and on the new therapies that are being developed for the treatment of SLE.

## B-cell target therapies

To date, the majority of studies have focused on B-cell target therapies ^4– 7^. Undoubtedly, B cells play a crucial role in the pathogenesis of SLE: their loss of tolerance, antigen presentation, autoantibody formation, stimulation of cytokine production, and T-cell activation have been identified as key players in the pathogenesis of SLE.

B cells are responsible for stimulating cytokine production, activating T cells, presenting self-antigens, and producing antibodies ^4– 7^. Therefore, biological therapies targeting and modifying the effects of B cells have been investigated in SLE and other autoimmune diseases. Available phase II and III trials of B-cell target therapies are summarized in Table 1.

**Table 1.** B-cell targeted biologic therapies in systemic lupus erythematosus (SLE).

Agent (mechanism of action)	Available evidence	Ongoing investigation
Rituximab (chimeric anti-CD20 moAb)	Primary endpoints were not met in LUNAR (SLE with lupus nephritis) and EXPLORER (SLE without non-nephritis) phase III trials. Promising results from the prospective RITUX study, investigating RTX as a steroid-sparing agent in lupus nephritis.	RITUXILUP trial (phase III) RTX as induction therapy followed by maintenance MMF (ClinicalTrials.gov Identifier: NCT01773616). The study has been terminated. (Study assessments for patients recruited continuing per protocol so patients receive a minimum of 6 months’ follow-up. No safety concerns have been raised.) RING study (phase III) Persistent proteinuria in lupus nephritis despite 6 months of standard immunosuppression (ClinicalTrials.gov Identifier: NCT01673295)
Belimumab (humanized anti- BLyS moAB)	Efficacy in the management of the musculoskeletal and hematologic manifestations of SLE (BLISS 52 and BLISS 76)	The BLISS-LN study is investigating the value of belimumab as an add-on therapy to standard care in the management of lupus nephritis (ClinicalTrials.gov Identifier: NCT01639339). CALIBRATE: RTX followed by belimumab compared with RTX and cyclophosphamide in the management of lupus nephritis (ClinicalTrials.gov Identifier: NCT02260934) EMBRACE: study of belimumab in ethnically diverse groups (ClinicalTrials.gov Identifier: NCT01632241)
Blisibimod (humanized anti- BLyS moAb)	PEARL-SC trial: a phase IIb study proved the efficacy, safety, and tolerability of blisibimod administration in SLE (ClinicalTrials.gov Identifier: NCT01162681)	Two trials (CHABLIS-SC1 and CHABLIS-SC2) are investigating the efficacy and safety of subcutaneous blisibimod in addition to standard therapy in SLE with and without nephritis (ClinicalTrials.gov Identifiers: NCT01395745 and NCT02074020).
Atacicept (TACI-Ig fusion protein)	ADDRESS II: a phase IIb, multi-center, randomized, double- blind, placebo-controlled, multi-dose, 24-week study to evaluate the efficacy and safety of atacicept in subjects with SLE (ClinicalTrials.gov Identifier: NCT01972568) Atacicept phase II/III in generalized systemic lupus erythematosus (APRIL-SLE) (ClinicalTrials.gov Identifier: NCT00624338)	Long-term safety and tolerability of atacicept (long-term follow-up of patients who participated in ADDRESS II)
Epratuzumab (humanized anti- CD22 moAb)	Study of epratuzumab versus placebo in subjects with moderate-to-severe general systemic lupus erythematosus (EMBODY 1) (ClinicalTrials.gov Identifier: NCT01262365) Study of epratuzumab versus placebo in subjects with moderate-to-severe general SLE (EMBODY 2) (ClinicalTrials.gov Identifier: NCT01261793)	Long-term safety and tolerability of epratuzumab
Tabalumab (humanized anti- BAFF moAB)	ILLUMINATE-1: a phase III, multi-center, randomized, double-blind, placebo-controlled study to evaluate the efficacy and safety of subcutaneous LY2127399 in patients with SLE (ClinicalTrials.gov Identifier: NCT01205438) ILLUMINATE-2: a 52-week, phase III, multi-center, randomized, double-blind, placebo-controlled study. Agent was effective at higher study dose.	Not applicable

Information regarding ongoing clinical trials in SLE was obtained from ClinicalTrials.gov. APRIL, a proliferation-inducing ligand; BAFF, B-cell-activating factor; BLyS, B-lymphocyte stimulator; CYC, cyclophosphamide; MMF, mycophenolate mofetil; moAB, monoclonal antibody; RTX, rituximab; TACI, tumor necrosis factor transmembrane activator and calcium modulator and cyclophilin ligand interactor; TNF, tumor necrosis factor.

### Rituximab

Rituximab is a chimeric monoclonal antibody (mAb) against CD20 receptors. CD20, or B-lymphocyte antigen CD20, is extensively expressed on immature, mature, and activated B cells but not on stem cells, plasma cells, or pro-B cells. Rituximab selectively binds CD20-positive cells and triggers a morphologic cellular change that ultimately results in B-cell depletion for 6 to 9 months in over 80% of patients ^8^. Rituximab is currently licensed for the treatment of non-Hodgkin’s lymphoma, chronic lymphocytic leukemia, antineutrophil cytoplasmic antibody (ANCA)-vasculitis, and rheumatoid arthritis (RA) ^9– 11^. To date, two randomized controlled trials have evaluated the efficacy and safety of rituximab versus placebo in patients with SLE: the EXPLORER trial (phase II/III evaluation of rituximab versus placebo in patients with moderately to severely active extra-renal SLE) ^12^ and the LUNAR trial (phase III trial evaluation of rituximab versus placebo in patients with class III or IV lupus nephritis) ^13^. Both trials hypothesized that adding rituximab to the standard of care of corticosteroids and immunosuppressants would control SLE activity better than the standard of care alone.

The EXPLORER trial recruited 257 patients (16–75 years old) with moderate or severe SLE. Participants had to fulfill four of the American College of Rheumatology criteria for SLE, including positivity for antinuclear antibodies (ANAs), an active disease at screening (defined as at least one domain with a British Isles Lupus Assessment Group [BILAG] disease activity index A score or at least two domains with a BILAG B), and a stable use of one immunosuppressive drug which was continued throughout the study. The effect of placebo versus rituximab in achieving and maintaining clinical response at week 52 was the primary endpoint.

The LUNAR study investigated the safety and efficacy of rituximab at 6 months as compared with placebo in addition to high-dose glucocorticoid (GC) and high-dose mycophenolate mofetil (MMF) (3g/day) in 144 patients with class III and IV lupus nephritis. The primary endpoint of the study was defined as the proportion of patients with complete or partial remission at 12 months. Complete response was defined as an improvement in serum creatinine from abnormal to normal levels or from normal to not more than 115% of baseline normal, a drop in the urine protein–creatinine ratio to less than 0.5, and the presence of urine sediment containing fewer than five red blood cells in a high-power field without casts at week 52. Neither trial demonstrated any significant difference between rituximab and placebo with regard to the primary and secondary endpoints.

Despite the negative results, some points are worth considering. First, biological therapies are currently taken into consideration for patients who are refractory to first-line conventional immunosuppressive therapies. A high percentage of patients in the two trials (especially in the EXPLORER trial) had no history of poor response to conventional therapies, which in itself could explain why the primary and secondary endpoints were not met. Furthermore, the efficacy of the biological therapy might have been masked by the concomitant high-dose GC therapy (up to 1 mg/kg) that was used in both trials. Lastly, the number of patients in the two studies (257 in the EXPLORER trial and 144 in the LUNAR trial) was smaller than in trials where the efficacy of other biological therapies in SLE was demonstrated.

The efficacy of rituximab in refractory disease has been reported in several observational studies involving SLE patients with renal and non-renal manifestations ^14– 22^. Moreover, a rituximab-based protocol (RA schedule) including methylprednisolone (500 mg on days 1 and 15) in the induction phase and MMF as a long-term maintenance treatment (Rituxilup trial) was recently proposed as a steroid-sparing regimen ^16^.

A different approach, initially employed as a rescue therapy in refractory lupus nephritis, has been proposed in an effort to minimize the long-term effects of both GCs and the immunosuppressive agents that are used for remission maintenance. This approach is based on intensified B-lymphocyte depletion consisting of four (weekly) plus two (monthly) doses of rituximab (375 mg/sm) in addition to two intravenous administrations of 10 mg/kg cyclophosphamide and three pulses of 15 mg/kg methylprednisolone followed by oral prednisone tapered to 5 mg/day in 10 weeks without further immunosuppressive maintenance therapy ^19, 22^. Our group, as well as others with considerable experience in this area, see rituximab as a therapeutic strategy for patients with refractory SLE although EXPLORER and LUNAR failed to achieve their endpoints.

With regard to safety, overall rituximab has been proven to be generally safe ^17– 22^. However, both early and long-term vigilance for infection post-infusion are important to further balance treatment risks and benefits. Besides, although hypogammaglobulinemia can be observed, not all patients who develop hypogammaglobulinemia are at increased risk of developing infection after B-cell-depleting therapy ^20^. A strict surveillance for side effects should be guaranteed, especially in the pediatric population with SLE, as infusion reaction and viral infections can occur. More rarely, severe cytopenia and central nervous system vasculitis can also be observed ^21^.

### Belimumab

Belimumab is a human immunoglobulin G1λ mAb that inhibits B-cell survival and differentiation by blocking the soluble B-lymphocyte stimulator (BLyS) ^23^. BLyS is a glycoprotein-based cytokine and a member of the tumor necrosis factor (TNF) family. It is an essential factor for controlling B-cell survival and is crucial for generating a normal immune response ^24^. There is sound proof that BLyS is overexpressed in patients with SLE and that its expression correlates with variations in disease activity ^25^. The role of B lymphocytes in the pathogenesis and clinical evolution of SLE supports the potential role of belimumab in the treatment of this condition.

Until 2005, when biological therapies became available, there were few trials on SLE (with the exception of lupus nephritis) as compared with those for other autoimmune diseases. Several observational studies had demonstrated the efficacy of belimumab for SLE treatment in all ethnic groups, including African-Americans ^26, 27^. However, belimumab was not approved or licensed by the US Food and Drug Administration (FDA) ( http://www.fda.gov) or the European Medicines Agency ( http://www.ema.europa.eu) for the treatment of active lupus until 2011 ^28^. This approval was a cornerstone for the treatment of SLE, since belimumab was the first drug to be licensed to treat lupus in over 50 years.

**Figure 2.**
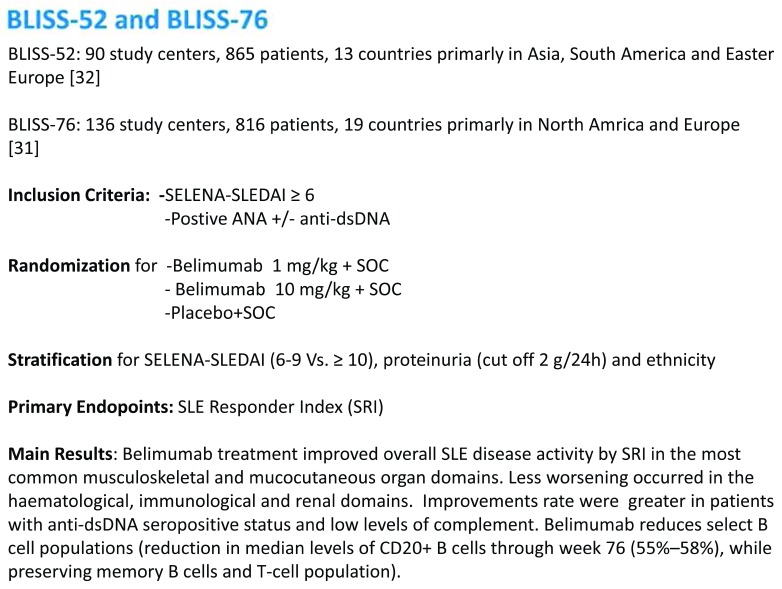
Main characteristics of phase III, randomized, placebo-controlled studies (BLISS 52 and BLISS 76) of belimumab, a monoclonal antibody that inhibits B-lymphocyte stimulator, in patients with systemic lupus erythematosus.

Phase I and II studies carried out in 2008 and 2009, respectively, provided initial support for its use. In total, 519 patients with mild to moderate SLE were recruited for these trials ^29, 30^. Results showed a safety profile for belimumab similar to that observed in the placebo group. However, these studies failed to show significant improvement in disease activity as compared with the placebo group. Two further trials (BLISS 52 and BLISS 76) were developed ^31, 32^. These international phase III trials enrolled a total of 1,684 patients with SLE (865 patients in the BLISS 52 trial and 819 in the BLISS 76 trial) with mild to moderate disease activity. Patients with central nervous system (CNS) involvement or renal involvement were excluded. A pooled analysis of BLISS 52 and BLISS 76 was performed to evaluate the efficacy and safety in the subpopulation of SLE patients with a more-severe disease activity score, defined by the BILAG domain score as follows: A (severe disease activity), B (moderate disease activity), or C (mild disease activity) in at least one of the domains at baseline. Patients with a SELENA-SLEDAI (Safety of Estrogens in Lupus Erythematosus - National Assessment–Systemic Lupus Erythematosus Disease Activity Index) score of more than 10, anti-double-stranded DNA (anti-dsDNA) of at least 30 IU/mL at baseline, or low complement relative to the normal range at baseline benefited more from the administration of belimumab than both the placebo arm and the SLE patients with a less-severe presentation ^33^. Figure 2 summarizes the key results of the BLISS 52 and BLISS 76 trials.

A further post-hoc analysis of the BLISS trials focused on the efficacy of belimumab on renal parameters in patients with renal involvement and in patients treated with MMF at baseline ^34^. The pooled analysis population consisted of 1,684 patients. Renal biomarkers showed improvement in baseline SELENA-SLEDAI renal involvement at week 52, especially in patients receiving MMF therapy. These data suggest that administering belimumab plus standard of care may benefit renal outcomes in patients with SLE.

There is an ongoing BLISS lupus nephritis phase III trial that hopefully will provide information regarding the safety and efficacy of belimumab and standard-of-care treatment in patients with active lupus nephritis ( https://clinicaltrials.gov/ct2/show/NCT01639339?term=belimumab+lupus+nephritis&rank=1).

Belimumab was administered intravenously in all of these trials. The BLISS 52 trial provided us with useful information regarding the efficacy and safety of subcutaneously administered belimumab. This randomized, double-blind, placebo-controlled trial involving 839 patients with active SLE as defined by a SELENA-SLEDAI score of at least 8 showed that subcutaneous administration significantly improved the SLE responder index (SRI) and decreased time to severe flare as compared with placebo plus standard of care. Furthermore, safety profiles in the two arms were similar ^35^.

Several other trials recently assessed the reliability and safety of the novel auto-injector for self-administration of subcutaneous belimumab 200 mg in patients with SLE ^36, 37^. The results suggest that the bioavailability of subcutaneously administered belimumab is similar to that of the intravenous administration and therefore may represent a valid treatment alternative.

### Atacicept

Atacicept is a human recombinant fusion protein containing both human IgG and the extracellular portion of the B-cell calcium-modulating ligand interactor (TACI) ^38^. Atacicept inhibits B-cell activation by blocking both BLyS and APRIL (a proliferation-inducing ligand) and consequently interrupts their signaling pathways involved in the proliferation of B cells ^39– 41^. APRIL is a secreted cytokine produced by a wide range of cells such as monocytes, dendritic cells, macrophages, and T cells that are involved in the immune response ^42, 43^.

Patients affected by SLE and other autoimmune disorders have higher BLyS and APRIL levels, thus suggesting that atacicept may be more efficient because of its dual blockade and its ability to target long-living plasma cells in addition to B cells ^39, 44^.

Preliminary results from *in vivo* models and two phase Ib trials showed that atacicept reduces both the number of B cells and circulating Ig levels with a minimal rate of adverse events ^38, 45– 47^.

On the basis of these preliminary studies, Ginzler *et al*. investigated the efficacy and safety of atacicept in patients with active lupus nephritis who were treated with high-dose steroids (up to 60 mg/day) and MMF (3 g/day) for 2 weeks ^48^. This trial was terminated early because of safety concerns, since three out of six patients developed severe hypogammaglobulinemia and severe infections ^48^. Whether atacicept was the culprit in these severe side effects remains a matter of debate ^49^.

APRIL-SLE was a later double-blind, placebo-controlled trial involving 461 patients with moderate to severe SLE who were randomly assigned to receive atacicept 75 or 150 mg subcutaneously. The primary endpoint of reducing flares (defined as BILAG A or B) was not met. A post-hoc analysis showed a beneficial effect in patients receiving 150 mg atacicept as compared with placebo. This preliminary observation is limited as a result of the premature discontinuation of the trial because of two infection-related deaths. When the relationship among treatment response, baseline biomarker levels, and treatment exposure is assessed, BLyS and APRIL may help to identify the patients who are most likely to benefit from atacicept treatment. However, the post-hoc analysis demonstrated that the infection rates were similar regardless of biomarker levels at baseline or at the time of atacicept exposure ^39^.

### Blisibimod

A number of studies reported overexpression of B-cell-activating factor (BAFF) in patients with SLE and a correlation between its serum levels and disease activity ^10^. Blisibimod is a subcutaneous BAFF inhibitor. B-cell survival and differentiation are highly dependent on BAFF ^50^. Its Fc domain is made up of human IgG and four BAFF-binding domain peptides that bind soluble and membrane-bound BAFF ^51^.

An initial placebo-controlled phase I trial proved that administering variable doses of blisibimod either by single injection or in four weekly doses led to a significant change in B-cell subpopulations: a decrease in naïve B cells and an increase in the number of memory B cells ^29^. In this study, the safety and tolerability profile of blisibimod in patients with SLE were comparable with those of placebo.

A follow-up phase II study, the PEARL-SC study, included 547 patients with SLE ^52^. All patients were positive for ANAs and anti-dsDNA antibodies and had a SELENA-SLEDAI score of at least 6 at baseline. Patients were randomly assigned to either placebo or subcutaneous blisibimod at one of three dose levels (100 mg once weekly, 200 mg once weekly, or 200 mg every 4 weeks) ^52^. High-dose blisibimod (200 mg once weekly) was particularly effective in patients with severe SLE, defined as a SELENA-SLEDAI score of at least 10, who were on GCs. Overall, the blisibimod group showed higher response rates than the placebo group, thus supporting the use of blisibimod as a therapeutic agent for patients with SLE.

An ongoing, randomized, double-blind, placebo-controlled phase III study—the CHABLIS-SC1—was presented at the European League against Rheumatism (EULAR) annual meeting in 2016. The purpose of the study is to evaluate the real impact of adding blisibimod to standard of care in patients with active SLE defined as a SELENA-SLEDAI score of at least 10 despite stable, ongoing corticosteroid therapy. Results from this trial are still being awaited ^53^.

### Tabalumab

Tabalumab is a human IgG4 mAb that binds and neutralizes both membrane and soluble BAFF ^54^. ILLUMINATE-1 and ILLUMINATE-2 are two phase III trials that were designed to evaluate the efficacy and safety of administering tabalumab subcutaneously in addition to standard of care in patients with active SLE ^54, 55^.

ILLUMINATE-1 was a 52-week, multi-center, randomized, double-blind, placebo-controlled study that enrolled 1,164 patients with moderate to severe SLE and a SELENA-SLEDAI score of at least 6. The primary endpoint (an SRI of 5 at week 52) was not achieved, but the response rates in the treatment group were higher than in the placebo group. Whether the high level of immunosuppression at baseline actually prevented the primary endpoint from being achieved is a valid argument. The secondary endpoints, which were defined as time to first severe SLE flare, GC-sparing effects, and changes in fatigue levels, were not met either. However, ILLUMINATE-1 results showed a significant decrease in anti-dsDNA levels in the tabalumab groups versus placebo as early as week 4 and up to week 52.

ILLUMINATE-2 was a 52-week, multi-center, randomized, double-blind, placebo-controlled study that enrolled 1,124 patients with active SLE ^40^. Its primary endpoint, which was defined as an SRI of 5, was met at week 52 by 38% of the patients receiving 120 mg tabalumab every fortnight in addition to standard of care compared with 27.7% in the placebo group. However, although a significant effect on anti-dsDNA reduction and C3 and C4 increase was observed in the tabalumab group, ILLUMINATE-2 did not meet its secondary endpoints. Furthermore, patients receiving tabalumab showed a decrease in the number of both total B cells and immunoglobulins. Tabalumab was more effective at achieving SRI-5 response in serologically active patients as compared with non-serologically active ones.

An additional analysis that focused on the impact on the kidney on the basis of the ILLUMINATE-1 and ILLUMINATE-2 trials demonstrated that, compared with placebo, tabalumab did not significantly affect the serum creatinine concentration, glomerular filtration rate, urine protein–creatinine ratio, or renal flare rates over 1 year in intent-to-treat or intent-to-treat plus urine protein–creatinine ratio patients.

### Ocrelizumab

Ocrelizumab is a second-generation anti-CD20 mAb and, like rituximab, is a B-cell-depleting agent. *In vitro* studies suggest that ocrelizumab may have a safer profile for complement activation and immunogenicity than rituximab as well as a lower frequency of both adverse infusion reactions and development of neutralizing anti-drug antibodies ^56, 57^.

BEGIN was a phase III randomized study that aimed to evaluate the efficacy and safety of ocrelizumab combined with a single, stable-background immunosuppressive medication and a corticosteroid regimen in patients with moderately to severely active SLE. The BEGIN study was terminated early because of the initial lack of response ^58^.

BELONG is a phase III randomized study. Its aim was to evaluate the efficacy and safety of ocrelizumab in patients with class III or IV lupus nephritis ^59^. Ocrelizumab was combined with either MMF or the Euro-Lupus Nephritis Trial regimen—cyclophosphamide followed by azathioprine (AZA) ^60^ —and a corticosteroid regimen. The BELONG trial was prematurely terminated because of the high rates of infection in the ocrelizumab arm. The infection rate was higher, especially in patients receiving background immunosuppressive therapy with MMF, which suggested a greater immunosuppressive synergy with ocrelizumab. The overall renal response among the 223 of the 381 patients who completed the 32-week period of treatment was not significantly higher than in the placebo group.

### Epratuzumab

Epratuzumab is a fully humanized mAb against CD22, a surface receptor expressed on mature B cells ^61^. CD22 is involved in B-cell activation and migration and has proven to be significantly overexpressed in patients with SLE ^62^.

Two randomized controlled trials of epratuzumab—ALLEVIATE-1 and -2—recruited patients with moderately to severely active SLE in order to evaluate health-related quality of life and corticosteroid use ^63^. Unfortunately, both trials were terminated because of disruption of the drug supply. Data analysis of the two studies at 12 weeks showed an increased response rate and improvement in the quality of life in the treatment arm albeit without reaching statistical significance.

EMBLEM was a phase IIb trial involving 227 patients with moderately to severely active SLE ^64^. The response rate of the treatment group was statistically significant as compared with the placebo group. A cumulative dose of 2,400 mg of epratuzumab resulted in significant clinical improvement.

EMBODY-1 and -2 were phase III trials that evaluated the efficacy of epratuzumab 600 mg every week or 1,200 mg every other week, administered in addition to standard of care ^65^. Neither study met the primary endpoint.

### Obinutuzumab

Obinutuzumab is a humanized, type II anti-CD20 antibody designed to increase direct cell death at the expense of reduced complement-dependent cytotoxicity activity ^66^. NOBILITY is an ongoing phase II trial that aims to evaluate the safety and efficacy of obinutuzumab in addition to MMF and corticosteroids in patients with class III or IV lupus nephritis ^67^.

### Other regimes

Ongoing research is paving the way for the use of synergetic approaches for B-cell immunomodulation. The CALIBRATE trial is investigating the effects of rituximab followed by maintenance therapy with belimumab in patients with refractory lupus nephritis (ClinicalTrials.gov Identifier: NCT02260934). Similarly, the ongoing SYNBIoSe trial (ClinicalTrials.gov Identifier: NCT02284984) is investigating the effects of combined therapy with anti-CD20 and anti-BLyS on SLE pathogenic autoantibodies. Results are anxiously awaited and may represent a cornerstone in the future management of patients with SLE.

## T-cell target therapies

Owing to the vast array of autoantibodies that are found, SLE has been typically classified as a “B-cell disease”. However, growing evidence supports the role of T cells in the pathogenesis of SLE and it is now widely accepted that SLE is a T-cell-driven disease ^68, 69^. T cells play a pivotal role in B-cell maturation, differentiation, antibody production, and class switching. A number of phenotypic and functional alterations have been identified in the T cells of patients with SLE, alterations likely to trigger the inflammatory response that is seen in these patients. New biological T-cell therapies, including cytokine production modulation and T-cell-mediated effects on B cells, represent a new therapeutic strategy for patients with SLE.

### Abatacept

Abatacept is a fusion protein composed of the Fc region of the immunoglobulin IgG1 fused to the extracellular domain of CTLA-4. It binds CD80 and CD86 with higher affinity than CD28 and blocks the co-stimulatory interaction between T and B lymphocytes, thus leading to unsuccessful T-cell activation and thereby preventing B-cell response ^70^.

Merrill *et al*. ^71^ carried out a randomized phase IIb trial and enrolled 118 SLE patients with polyarthritis, discoid lesions, or pleuritis or pericarditis (or both). The primary and secondary endpoints of the study were not met, and after more than 12 months there were no significant differences in (BILAG A/B) flare rates between the abatacept and placebo groups. Interestingly, post-hoc analyses revealed that severe flares (BILAG A) were less frequent in the abatacept group compared with the placebo group ^72^.

Furie *et al*. ^73^ conducted a 12-month, randomized, phase II/III, double-blind study that enrolled 298 SLE patients with active class II or IV lupus nephritis and that added abatacept to MMF and GCs. No differences among treatment arms were observed in the time to confirmed complete response or in subjects with confirmed complete response following 52 weeks of treatment.

Although the primary endpoints of these studies were not met, further evidence supports the potential efficacy of abatacept in SLE ^74^ and highlights that its role as a therapeutic alternative has yet to be fully defined.

### Laquinimod

Laquinimod (5-chloro-N-ethyl-4-hydroxy-1-methyl-2-oxo-N-phenyl-1,2-dihydroquinoline-3-carboxamide) is an immunomodulatory drug that alters both lymphocytes and monocyte/macrophages in murine experimental autoimmune models ^75^. It has been used successfully in clinical trials in patients with multiple sclerosis with a mild adverse-event profile ^70^. Laquinimod downregulates pro-inflammatory cytokines—interleukin-6 (IL-6), IL-17, IL-23, and TNF-α—and increases the production of IL-10, thereby exerting an immunomodulatory effect on antigen-presenting cells that target T cells. Its effects lead to immunomodulation in favor of T helper 1 over T helper 2 cells ^75^.

In their phase IIa study, Jayne *et al*. (ClinicalTrials.gov Identifier: NCT01085097) (https://clinicaltrials.gov/ct2/show/NCT01085097) evaluated laquinimod in combination with MMF and GCs and analyzed its efficacy and safety in 46 patients with active lupus nephritis. The preliminary results of the trial seem promising and include improvement in both renal function and proteinuria in patients treated with laquinimod, and there was no evidence of any increased frequency of side effects.

### Edratide

Edratide is a tolerogenic peptide based on the sequence on the first complementarity-determining (CDR1) region of anti-DNA mAb (16/6 idiotype) ^71^. Preliminary results of the studies were promising and showed that edratide downregulates IL-1β, IFN-γ, and IL-10 and upregulates transforming growth factor-beta (TGF-β), thus reducing the production of BLyS ^41, 76^. A 24-week phase II trial that enrolled 340 patients with SLE failed to meet its primary endpoints, defined as a reduction of both SLEDAI-2K and mean SLEDAI, and therefore the trial was prematurely discontinued ^77^. Further analysis showed that the secondary endpoint, which was an improvement in BILAG scores, was met in the 0.5 mg edratide group and showed a statistical difference compared with the placebo arm ^77^.

### Rigerimod/Lupuzor

Rigerimod, which is also known as Lupuzor, is a peptide derived from a region of the U1-70k snRNP protein, a nuclear riboprotein and spliceosome component ^78^. The mechanism of action of rigerimod is not fully understood, but preliminary studies showed that it acts as an immunomodulator by binding major histocompatibility complex (MHC) class II and consequently inhibiting T-cell reactivity and restoring immune tolerance ^79^.

The safety and efficacy of rigerimod were assessed in a phase IIa trial that enrolled 20 patients with active SLE. Patients were treated with two weekly subcutaneous injections of rigerimod (200 µg). A reduction in physician-assessed disease activity was observed, as was a decrease in anti-dsDNA levels ^80^. A further, randomized, placebo-controlled phase IIb trial confirmed these preliminary results. In fact, 136 out of the 149 SLE patients who were enrolled in the trial showed a significant reduction in clinical SLEDAI between baseline and week 12 as compared with placebo ^80^. Further studies are ongoing.

## Immunoregulatory molecule-targeting therapies

### Proteasome inhibitors: bortezomib

Bortezomib is a proteasome inhibitor that is currently approved for the treatment of multiple myeloma. Bortezomib reversibly binds to the 26S proteasome and inhibits its chymotrypsin-like activity, resulting in plasma cell depletion ^81^. Recently, Alexander *et al*. ^82^ investigated the safety and efficacy of bortezomib in 12 patients with refractory SLE. Although a subgroup of patients showed a decrease in proteinuria, the majority of patients had to discontinue treatment because of the high incidence of adverse events. Further studies are needed and must include next-generation proteasome inhibitors with a greater tolerability profile.

### Interleukin-6-targeting therapies

IL-6 is a pleiotropic cytokine with a wide range of biological activities that plays a crucial role in immunoregulation and inflammation. IL-6 promotes B-cell maturation and antibody production and contributes to a plethora of immune cell activities, such as cell activation, proliferation, differentiation, and cytokine secretion. IL-6 acts in concert with IL-1β and TNF-α to drive inflammation and stimulates the differentiation of potent inflammatory Th17 cells and B-cell differentiation into plasma cells. In addition, it is key for certain homeostatic mechanisms as well as the acute-phase response.

*In vitro* and *in vivo* studies have shown high levels of IL-6 in SLE. A reduction in anti-dsDNA antibodies has been observed in murine studies after blocking the IL-6 cascade ^83^.

***Tocilizumab.*** Tocilizumab is a humanized mAb directed against IL-6 receptors. Recently, Illei *et al*. ^84^ enrolled 16 SLE patients with mild to moderate disease activity in an 8-week, phase I dosage-escalation study to investigate the efficacy and safety of tocilizumab. Disease activity showed significant improvement, including a decrease in the SELENA score in eight of the 15 enrolled patients and a concomitant, significant decrease in anti-dsDNA titers. However, tocilizumab treatment led to dosage-related transient decreases in the absolute neutrophil count, resulting in the withdrawal of one of the patients. Further studies are needed to establish the efficacy and recommendations of tocilizumab for the treatment of SLE.

***Sirukumab.*** Sirukumab is a humanized mAb that binds to IL-6 and consequently inhibits its biological activity. Szepietowski *et al*. ^85^ investigated the safety and efficacy of sirukumab in a phase I, double-blind, placebo-controlled study involving 15 patients with SLE. Adverse events were observed more often in the sirukumab group than in the placebo group (90% versus 80%). Sirukumab led to sustained, dose-independent decreases in white blood cell counts, absolute neutrophil counts, and platelet counts and minor increases in total cholesterol levels. No differences in clinical efficacy were observed between the sirukumab arm and the placebo group. A recent multi-center, randomized, double-blind study was set up to assess the efficacy and safety of sirukumab in 25 SLE patients with class III or IV active lupus nephritis receiving concomitant immunosuppressive therapy ^86^. Six patients discontinued the study early, five of whom had infection-related adverse events. The median percentage change in proteinuria from baseline to week 24 in the sirukumab arm was 0%. In the sirukumab group, 47.6% of patients experienced at least one severe adverse event by week 40, most of which were infection related. No deaths or malignancies occurred. This study failed to demonstrate an acceptable safety profile.

### Interferon-alpha-targeting therapies

Recent studies have brought to light the role of the activation of the type I IFN pathway in the cells of patients with SLE. In fact, type I IFN pathway activation is associated with significant clinical manifestations of SLE and the presence of autoantibodies specific for RNA-binding proteins ^87^. IFN-α-targeting therapies include sifalimumab, rontalizumab, IFN-α kinoid, and anifrolumab.

***Sifalimumab.***Sifalimumab is a human IgG1 mAb that binds IFN-α. Preliminary phase I studies have provided encouraging results and have shown that sifalimumab tends to reduce the number of disease flares ^88, 89^. The efficacy and safety of sifalimumab were assessed in a phase IIb, randomized, double-blind, placebo-controlled study involving 431 adults with moderately to severely active SLE. Patients received monthly intravenous administrations of sifalimumab (200, 600, or 1,200 mg), and a high percentage of all dosage arms showed index response and clinical improvement at week 52 ^90^.

***Rontalizumab.***Rontalizumab is a human IgG1 mAb that binds all known isoforms of human IFN-α. McBride *et al*. ^91^ ran a preliminary safety, pharmacokinetic profile, and pharmacodynamic effect trial of rontalizumab in a cohort of 60 patients with SLE in a dose-escalation study. More recently, Kalunian *et al*. ^92^ conducted a phase II study in patients with active SLE treated with 750 mg intravenous rontalizumab every 4 weeks or placebo and with 300 mg subcutaneous rontalizumab every 2 weeks or placebo. Although the primary and secondary endpoints of this trial—reduction in disease activity at week 24 by BILAG (primary) and SRI (secondary)—were not met, an exploratory analysis showed that rontalizumab treatment was associated with an improvement in disease activity, reduced flares, and decreased corticosteroid use in patients with SLE with low IFN signature.

***Interferon-alpha kinoid.*** IFN-α kinoid is a drug consisting of inactivated IFN-α coupled with a carrier protein (that is, keyhole limpet hemocyanin). IFN-α kinoid is an IFN-α immunogen, which, when appropriately adjuvanted, induces transient neutralizing antibodies but no cellular immune response to the cytokine and which apparently causes no side effects ^93^. Recently, Lauwerys *et al*. ^94^ examined the safety, immunogenicity, and biologic effects of active immunization with IFN-α kinoid in 28 patients with mild to moderate SLE in a randomized, double-blind, placebo-controlled, phase I/II dose-escalation study. Although IFN-α kinoid was well tolerated, no difference in disease activity was reported between groups.

***Anifrolumab.*** Anifrolumab is an antagonist human mAb that targets IFN-α receptor 1 (IFNAR1). Recently, Merrill *et al*. ^95^ evaluated anifrolumab (300 mg, 1,000 mg every 4 weeks for 1 year) in a randomized, phase IIb study that enrolled 305 SLE patients with moderate to severe disease activity. Compared with placebo, anifrolumab treatment resulted in higher rates of improvement in multiple organs, showing the greatest impact with the administration of 300 mg anifrolumab. It is noteworthy that the majority of patients had baseline involvement of the mucocutaneous or musculoskeletal domains (or both) of SLEDAI-2K and BILAG. Patients in the 300 mg arm who had positive anti-dsDNA or low complement levels (or both) showed lower scores at day 365. However, among patients who had normal anti-dsDNA or normal complement levels (or both) at baseline, a slightly higher number of patients treated with 300 mg developed new anti-dsDNA or hypocomplementemia as compared with baseline. Currently, at least three ongoing phase III clinical trials are evaluating the efficacy and safety of anifrolumab versus placebo in patients with moderately to severely active autoantibody-positive SLE while receiving standard-of-care treatment (ClinicalTrials.gov Identifiers: NCT02446899, NCT02446912, and NCT02794285).

### Interferon-gamma target therapies: AMG811

IFN-γ is a pro-inflammatory cytokine that modulates the immune system, including B cells, T cells, and macrophages. Although some of the above-reported studies provide evidence on the effect of blocking type I IFNs in SLE, only few have investigated the potential effect of blocking type II IFNs.

AMG 811 is a human IgG1 mAb that selectively targets and neutralizes human IFN-γ. Martin *et al*. ^96^ enrolled 28 SLE subjects with active lupus nephritis being treated with AMG 811 in addition to MMF or AZA. The study reported no significant difference in disease activity, but a higher rate of infections was observed in the drug arm. Further studies are needed to assess the efficacy of the blockade of type II IFN.

### Abetimus sodium (LJP-394)

Abetimus sodium is a tetrameric oligonucleotide that was specifically designed to decrease anti-dsDNA antibody levels. In fact, abetimus sodium cross-links anti-dsDNA antibody receptors on their cell surface, triggering the signal transduction pathways, thus inducing B-cell anergy or apoptosis ^97^. Preliminary results showed a significant and persistent decrease in anti-dsDNA titers in patients with SLE, and there was no increase in adverse events ^98^. Based on these promising results, a 76-week, double-blind, placebo-controlled study that enrolled 230 SLE patients, including patients with lupus nephritis, was set up to investigate LJP-394 efficacy. The trial showed a significant decrease in renal flares and lengthened the time to renal flare to 76 weeks in a subset of patients with high-affinity serum IgG fraction for the DNA epitope of LJP-394. However, these results were not confirmed in the following phase III studies (ASPEN trials), which enrolled 317 and 943 patients with SLE, respectively, and did not meet their primary endpoints ^99, 100^.

### Anti-TNF-α target therapies

The role of TNF-α has recently shifted from being a pro-inflammatory cytokine to an immunoregulatory molecule that can alter the balance of regulatory T cells ^101^. Anti-TNF-α target therapies are effective for managing chronic inflammatory disorders such as moderate-to-severe RA and Crohn’s disease ^102– 104^. However, their potential role in SLE is still controversial, since their use has been associated with new or aggravated forms of autoimmunity such as the formation of autoantibodies, including ANAs, anti-dsDNA antibodies, and anticardiolipin antibodies ^105^. To date, experience on the use of TNF inhibitors, such as etanercept and infliximab, is limited in SLE as it has been associated with the induction of anti-dsDNA; however, anti-TNF antibody could be of potential therapeutic benefit for a selected subgroup of patients with SLE (for example, SLE arthritis). Evidence on the use of newer anti-TNF agents (for example, certolizumab) in patients with SLE is anecdotal ^105^.

***Etanercept***. Etanercept is a human TNF receptor p75 Fc fusion protein. It is a dimer of a chimeric protein that is genetically engineered by fusing the extracellular ligand-binding domain of human TNF receptor-2 to the Fc domain of human IgG1 ^106^. Etanercept competitively inhibits TNF binding to the cell surface TNF receptor. A randomized, double-blind, placebo-controlled, phase II trial recently investigated the potential use of etanercept for the treatment of lupus nephritis ^107^. The study was terminated early (with just one enrolled subject) because of potential safety issues. An ongoing trial is investigating the potential use of intradermal etanercept for the treatment of discoid lupus erythematosus ^108^.

***Infliximab.*** Infliximab is a human-murine chimeric mAb directed against TNF-α ^109^. A recent trial investigated the potential use of infliximab in patients with active class V lupus nephritis ^110^; however, it was terminated early because of failure to recruit patients with membranous lupus nephritis who had never been treated with AZA.

### Sphingosine 1-phosphate receptor type 1 agonist (KRP-203)

Sphingosine 1-phosphate (SP1) is a pleiotropic lipid mediator that is involved in the regulation of a broad spectrum of cellular functions, including proliferation and survival, cytoskeletal rearrangements, cell motility, and cytoprotective effects ^111, 112^. Following the promising preliminary results on the use of KRP-203 (an agonist of SP1) in animal models ^113, 114^, an ongoing phase II trial is currently evaluating the safety and efficacy of KRP-203 in patients with subacute cutaneous lupus erythematosus (ClinicalTrials.gov Identifier: NCT01294774).

### JAK inhibitors

Jaks are tyrosine kinases (Jak1, Jak2, Jak3, and Tyk2) that bind to cell receptor subunits and mediate the intracellular signaling initiated by IFN, many interleukins, colony-stimulating factors, and hormones such as prolactin, erythropoietin, and growth hormone. Following receptor ligation, Jak becomes activated and phosphorylates the latent transcription factors known as signal transducers and activators of transcription (STATs). Then STATs, in homodimers or heterodimers, translocate into the nucleus where they regulate gene transcription. Mutations of Jak or STAT in humans are associated with severe immune dysfunction, revealing the fundamental role of this pathway in the induction and regulation of immune responses ^115– 119^. Tofacitinib, a small molecule that inhibits Jak3, Jak1, and (to a lesser degree) Jak2, has been proven efficacious in RA in phase III trials, and ruxolitinib, which inhibits Jak2, was approved by the FDA to treat myelofibrosis ^120– 122^. Notably, a series of Jak-STAT signaling cytokines, especially IFN-Is, IL-10 and IL-6, as well as the hormone prolactin, have been implicated in the pathogenesis of SLE ^123– 126^. In this context, targeting the Jak-STAT pathway has emerged as an attractive approach to manage inflammation and auto-immunity in SLE. Treatment of lupus-prone mice with JAK2 inhibitors led to prevention or improvement of established disease. In MRL/lpr mice, administration of tryphostin AG490 from 12 to 20 weeks of age led to a decrease in proteinuria, T-cell and macrophage infiltrates, expression of IFN-γ, serum level of dsDNA, and deposition of IgG and C3 in the kidney ^127^. A disease prevention protocol with another Jak2 inhibitor, CEP-33779, which was started at the age of 8 weeks up to 21 weeks, prevented the development of nephritis. In addition, administration of CEP-33779 in NZB/W F1 mice with established nephritis was proven superior to treatment with dexamethasone and cyclophosphamide, resulting in improved survival, reduced proteinuria, decreased dsDNA antibodies, and decrease in the autoantibody-producing spleen plasma cells. Finally, several cytokines associated with SLE pathogenesis, including IL-12, IL-17A, IL-6, IL-4, and TNF-α, were also downregulated upon treatment with the Jak2 inhibitor ^128^.

## Future therapeutics

Several new agents, including an anti-Fcγ-receptor-IIb, Toll-like receptor inhibitors, Jak inhibitors, kinase inhibitors specifically targeting spleen tyrosine kinase or phosphoinositide-3-kinase, and histone deacetylase inhibitors, are currently under investigation as candidate therapies for SLE ^78^. The rationale for targeting such pathways arose from the growing body of evidence investigating monogenic disorders with a lupus-like phenotype. These can be organized into physiologic pathways that parallel mechanisms of disease in SLE. Examples include genes important for DNA damage repair (for example, TREX1), nucleic acid sensing and type I IFN overproduction (for example, STING and TREX1), apoptosis (FASLG), tolerance (PRKCD), and clearance of self-antigen (DNASE1L3). Further study of monogenic lupus may lead to better genotype/phenotype correlations in SLE. Eventually, the ability to understand individual patients according to their genetic profile may allow the development of more targeted and personalized approaches to therapy ^128– 130^.

## Discussion

The pathogenesis of SLE is yet to be fully understood, and new biological therapeutic options directed against molecular mediators of SLE are being developed now that we are reaching a better understanding of the pathogenic pathways and the cellular and molecular mediators underlying SLE. Novel biological treatments are rapidly emerging; however, further investigational studies are needed. The road to new therapeutic options for the treatment of SLE is still challenging. Table 2 summarizes early terminated studies or available investigation on drugs whose use in SLE is still speculative at this stage (for example, ustekinumab, a mAb directed against the p40 subunit of IL-12/IL-23, has been FDA approved to treat psoriasis and is now in trials for SLE).

**Table 2.** Other targeted biologic therapies in systemic lupus erythematosus (SLE).

Agent (mechanism of action)	Available evidence	Ongoing investigation
AMG 557 (inducible T-cell co- stimulator ligand, or ICOSL)	Phase 1b, randomized, double-blind, placebo- controlled study, patients received AMG 557 210 mg (n=10) or placebo (n=10) weekly for 3 weeks and then every other week for 10 additional doses in patients with SLE and active lupus arthritis (ClinicalTrials.gov Identifier: NCT01683695)	N/A
BG9588 (anti-CD40 ligand antibody)	Twenty-eight patients with active proliferative lupus nephritis were scheduled to receive 20 mg/kg of BG9588 at biweekly intervals for the first three doses and at monthly intervals for four additional doses. The study was terminated prematurely because of thromboembolic events (ClinicalTrials.gov Identifier: NCT00001789).	N/A
IL-2 treatment	Treatment with low-dose recombinant human IL-2 selectively modulated the abundance of regulatory T (Treg) cells, follicular helper T (T FH) cells, and IL-17- producing helper T (TH 17) cells, but not TH 1 or TH 2 cells, accompanied by marked reductions of disease activity in patients with SLE.	- Low-dose IL-2 for treatment of SLE (Charact-IL-2) (ClinicalTrials.gov Identifier: NCT03312335) - Low-dose IL-2 treatment in SLE (ClinicalTrials.gov Identifier: NCT02084238) Induction of regulatory T cells by low-dose IL-2 in autoimmune and inflammatory diseases (ClinicalTrials.gov Identifier: NCT01988506)
Ustekinumab (mAb directed against the p40 subunit of IL-12/IL-23)	Anecdotal case reports in patients with SLE and psoriasis	A phase 2a, efficacy and safety study of ustekinumab in SLE (ClinicalTrials.gov Identifier: NCT02349061)

IL, interleukin; mAb, monoclonal antibody; N/A, not applicable.

Assessment studies must be carried out for all novel drugs in order to evaluate their efficacy, safety, and immunogenicity profiles. Despite the evident difficulties, phase II and III studies should be standardized and efficacy endpoints need to be properly defined and customized to a specific SLE population, to specific clinical manifestations, and to organ involvement. Post-marketing surveillance and registry data are also fundamental when evaluating the long-term safety, efficacy, and cost-effectiveness of these novel therapies. Therefore, once they have been developed, new biological treatments must undergo a long and expensive process before becoming real candidates as SLE primary therapeutic options.

A careful analysis of failed trials is also highly recommended. Understanding why these studies have been unsuccessful is fundamental in order to build better-standardized trial methods and to assess whether further studies are warranted to investigate a given drug.

Standard of care needs to be considered when designing a trial. It is difficult to achieve a significant difference between the placebo and drug treatment arms when patients are receiving standard-of-care treatment. For example, GC use can increase the response rate in the placebo group and thereby influence trial results. Therefore, the exposure to and dosage of GC should be limited. In the context of disease activity, the use of GCs should be adjusted and the GC dosage should be balanced between arms to minimize introduced bias. Ideally, for mild lupus manifestations, GCs should be omitted if possible. Drug trials focusing on patients with dermal and musculoskeletal SLE manifestations might demonstrate results of experimental therapy more clearly if they omit GC use as a standard of care. However, with the available level of evidence, this strategy would be unethical to implement for patients with moderate-to-severe lupus.

As we acquire a better understanding of SLE pathogenesis, we may be able in the near future to correlate clinical manifestations of SLE with specific pathogenic pathways (that is, cellular and molecular mediators that could be specifically targeted). Therefore, biological therapies could become even more important in SLE management. We hypothesize that, in the future, the management of SLE could be tailored to the patient’s specific pathogenic manifestations, genetic background, and clinical characteristics by the use of specific biological therapies.

In conclusion, there is a great deal of enthusiasm regarding the use of new immunomodulatory therapies in SLE. Newly available evidence is guiding us toward an understanding of the molecular and cellular pathways that are involved in the immunopathogenesis of the disease, thus leading the way to new targeted therapies.
